# Effects of thresholding on correlation-based image similarity metrics

**DOI:** 10.3389/fnins.2015.00418

**Published:** 2015-10-29

**Authors:** Vanessa V. Sochat, Krzysztof J. Gorgolewski, Oluwasanmi Koyejo, Joke Durnez, Russell A. Poldrack

**Affiliations:** ^1^Poldrack Lab, Department of Psychology, Stanford UniversityStanford, CA, USA; ^2^Program in Biomedical Informatics, Stanford UniversityStanford, CA, USA; ^3^Department of Data Analysis, Ghent UniversityGhent, Belgium

**Keywords:** neuroimaging, functional magnetic resonance imaging, image comparison, thresholding, image classification, human connectome project

## Abstract

The computation of image similarity is important for a wide range of analyses in neuroimaging, from decoding to meta-analysis. In many cases the images being compared have empty voxels, but the effects of such empty voxels on image similarity metrics are poorly understood. We present a detailed investigation of the influence of different degrees of image thresholding on the outcome of pairwise image comparison. Given a pair of brain maps for which one of the maps is thresholded, we show that an analysis using the intersection of non-zero voxels across images at a threshold of *Z* = ±1.0 maximizes accuracy for retrieval of a list of maps of the same contrast, and thresholding up to *Z* = ±2.0 can increase accuracy as compared to comparison using unthresholded maps. Finally, maps can be thresholded up to to *Z* = ±3.0 (corresponding to 25% of voxels non-empty within a standard brain mask) and still maintain a lower bound of 90% accuracy. Our results suggest that a small degree of thresholding may improve the accuracy of image similarity computations, and that robust meta-analytic image similarity comparisons can be obtained using thresholded images.

## 1. Introduction

The computation of similarity between images is an increasingly important component of neuroimaging analyses. In the context of reproducibility, statistical brain maps must be compared to evaluate if a new result has successfully replicated a previous one. For approaches that involve clustering, a distance or similarity matrix is commonly defined that makes a comparison between all pairwise maps. For meta-analytic decoding (Yarkoni et al., 2011), one must be able to identify similarity between the target image and each image in the relevant database.

One challenge in computation of image similarity is the presence of empty (zero-valued) voxels due to thresholding. The clearest example comes from coordinate-based meta-analysis, where voxels outside of regions with activation peaks will have a zero value. However, the problem arises in other domains as well, such as the NeuroVault database (Gorgolewski et al., 2013, 2015). The maps in NeuroVault represent a broad range of statistical tests, and while a warning is issued when a user uploads a thresholded map, there is no hard restriction. At the time of our analysis, for the 774 publicly available maps in the NeuroVault database, 60 (~7.7%) had fewer than 25% of non-empty voxels observed within an MNI template brain mask. Recently, NeuroVault has implemented the ability to compare a single result map to all others in the database. This reality presents with the challenge of performing image comparison in the presence of thresholding choices that may introduce many “faux zeros,” or even eliminate negative values completely. The impact of these empty voxels on image comparison is not currently understood.

In the present work we examine the effects of thresholding on image similarity metrics. Specifically, we test the accuracy of classifying brain images according to their experimental contrast, using several levels of image thresholds and strategies to handle values that are rendered empty by thresholding. We approach the problem from a machine learning framework, assessing the accuracy of classifying image contrasts at the varying levels of thresholding. The results demonstrate that limited thresholding may in some cases have a beneficial effect on classification accuracy, and that accurate classification can be retained even under fairly aggressive thresholding.

## 2. Materials and methods

**Figure 1:**
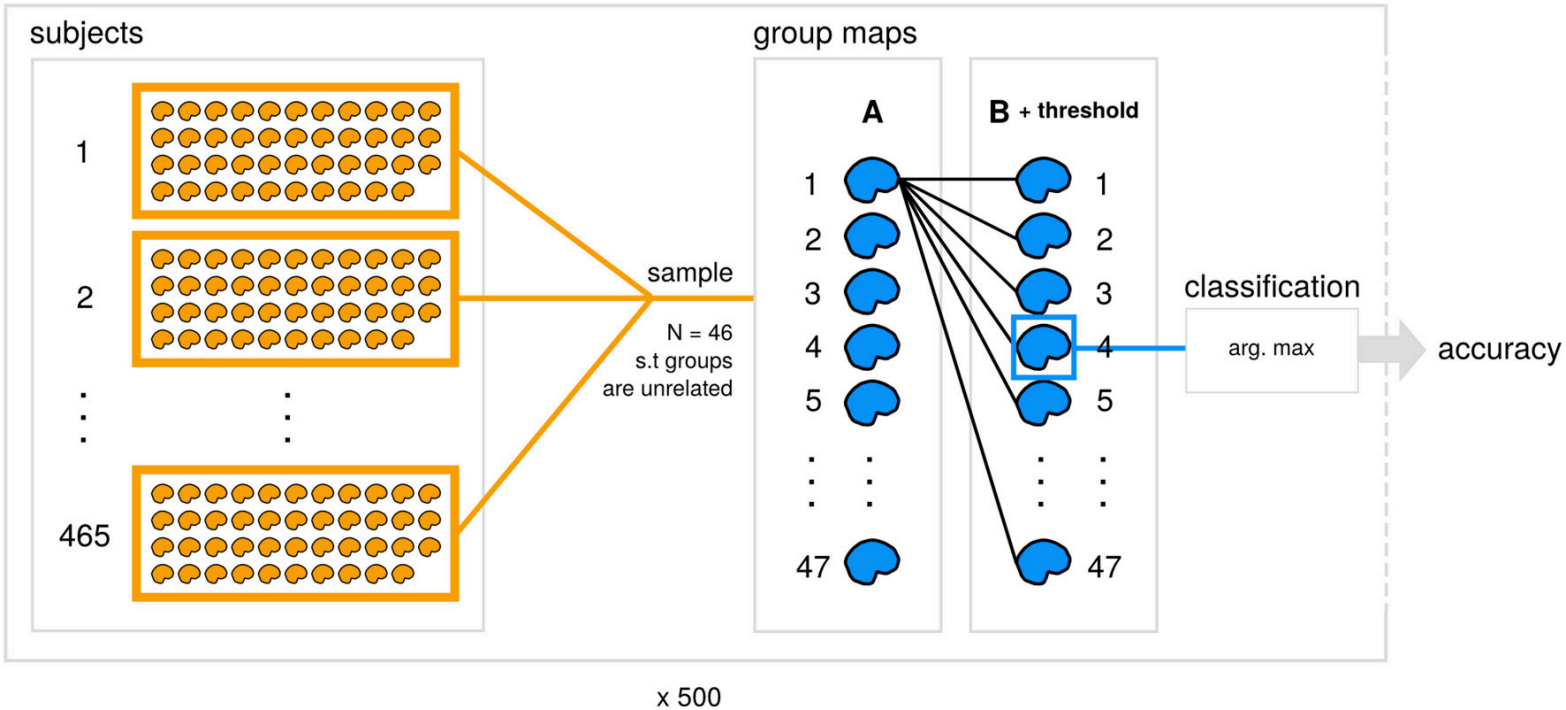
**Data generation and analysis process**. A subset of 465 datasets from the Human Connectome Project (subjects) is used to generate 47 contrast maps (group maps) for each of groups A and B for 500 subsamples. Within each subsample, an unthresholded image from A is compared with each thresholded image from B with a particular similarity metric and comparison strategy applied. Each image from A is then assigned the predicted class for the max. arg from the set of B, and accuracy is calculated for the subsample.

The code and results for all analyses reported here is available (https://github.com/vsoch/image-comparison-thresholding), and a summary of our classification framework is detailed in Figure 1.

### 2.1. Data

To generate a large set of group maps across many behavioral contrasts, we utilized images from the Human Connectome Project (HCP; Van Essen et al., 2012, 2013). The HCP provides access to large datasets of brain images, including a data release of 501 subjects (including relatives) with the majority having completed seven functional tasks (Van Essen et al., 2013). The large number of subjects and assortment of functional paradigms allowed us to generate samples of unrelated individuals for a wide range of contrasts, and assess the influence of levels of thresholding (Section 2.4 Empty voxels in brain statistical maps) combined with a strategy for handling said empty voxels (Section 2.5 Strategies to handle empty voxels) in a classification framework. Specifically, we are studying the influence of image thresholding and the choice of how to handle non-overlapping voxels on the ability to match any given contrast from one group to the equivalent contrast in a second group.

To generate two groups, A and B, to be used in our random subsampling procedure (Section 2.6 Assessing influence of thresholding on classification accuracy), we first subset the HCP data to the 465 out of 501 subjects that have data for all contrasts across all tasks. For each of 500 iterations, we used a random sampling procedure to generate two groups (*N* = 46) for comparison that ensured no related subjects between groups. To accomplish this, we take a random sample of 46 subjects for group A, and randomly sample from the remaining subjects, adding to group B only in the case that the sample has no relations to individuals in group A. We repeat this procedure until we have amassed an appropriately sized sample.

### 2.2. Contrast selection and statistical map generation

We first filtered the contrasts to a unique subset. Across the seven functional tasks from HCP (emotion, working memory, relational, gambling, language, social, and motor), there were a total of 86 contrasts (6, 30, 6, 6, 6, 6, and 26 respectively for each task), and we filtered this set down to 47 (3, 19, 3, 3, 3, 3, 13 respectively) to remove redundancy in the maps, including negation of contrasts and inverse subtractions (e.g., “faces - shapes” vs. “shapes - faces”). The list of contrasts is available in Supplementary Data 1. Single subject data for these contrasts was used to derive group maps for comparison; for each group/contrast, a one-sample *t*-test was performed using the FSL randomise tool, which returns a whole-brain t-statistic map. This procedure resulted in 47 whole-brain, unthresholded t-statistic maps for each of two unrelated groups, A and B, for each of 500 iterations. We normalized these maps to Z-scores using an implementation of Hughetts transform (https://github.com/vsoch/TtoZ) that has better precision than the tools currently employed in standard neuroimaging software packages Hughett, 2007.

### 2.3. Similarity metrics

While choice of a similarity metric is just as important as a strategy for handling empty voxels, for the purposes of this study we chose two commonly utilized metrics, Pearson's R correlation coefficient, and Spearman's Rank correlation coefficient (Taylor, 1895), implemented with “pearsonr” and “spearmanr” in the python package scipy (Jones et al., 2014).

### 2.4. Empty voxels in brain statistical maps

As discussed previously, image thresholding introduces empty voxels in brain statistical maps. We define a set of thresholds, T, ranging from 0.0 (no threshold applied) to ±13.0 in increments of 1.0 to cover the entire range of possible Z-Scores defined for the images (minimum = −12.27, maximum = 11.18). We consider two separate analyses: first to include positive and negative values, and second to include only positive values, as researchers interested in positive associations alone may completely eliminate negative values from a map. In the case of including positive and negative values for a given threshold, T, the images were thresholded to only include values above +T, and below −T.

### 2.5. Strategies to handle empty voxels

The default of most software is to take one of two approaches: replacing empty voxels with 0, or eliminating them entirely from the comparison set. We chose these two strategies for handling empty voxels to mirror this practice. We first consider data that is only complete, “complete case analysis” (CCA). This means an intersection-based strategy that limits the comparison set to the intersection of non-zero, non-NaN voxels between two images. Second, we consider the case of single-value imputation (SVI), where empty/NaN values are replaced with zeros. Each of these two strategies was applied to each of two images for comparison.

### 2.6. Assessing influence of thresholding on classification accuracy

#### 2.6.1. Extraction of pairwise scores

Within each iteration, we calculated pairwise Pearson and Spearman scores for each of the 47 contrasts for group A (unthresholded) against all 47 contrasts for group B with a particular strategy for handling empty voxels (CCA and SVI) and threshold applied. Note that because CCA excludes any voxels not present in both images, it is equivalent to thresholding each map using the non-zero voxels shared between an unthresholded image A and thresholded image B. For each of our 14 thresholds (including a level of 0.0 that is equivalent to no thresholding applied), we tested the comparisons using both positive and negative values, and only positive values. Using the MNI standard template brain mask (2 mm), a completely unthresholded image would allow for 228, 483 voxels for comparison. In the cases of no non-zero values surviving a level of thresholding, no overlapping finite values, or having fewer than three voxels from which to compute a score, we assert that the maps cannot be compared and thus have no similarity, and ascribe a score of NaN.

#### 2.6.2. Assessment of empty voxels on classification accuracy

Within each of the 500 subsamples, we make the basic assumption that equivalent contrasts defined between groups A and B should be most similar, meaning that a particular contrast for group A should have the greatest similarity with the equivalent contrast for group B across all contrasts. We can make this assessment for each strategy to handle empty voxels, across all thresholds, and calculate a mean accuracy for each strategy, threshold, and metric. Specifically:

**     For each of 500 subsamples:**
         Subset data to unrelated groups A and B
         For each unthresholded map, A_*i*_ in A
              Apply each threshold in *Z* = ±0:13, and *Z* = + 0:13 to
              all of B
              Calculate similarity for each of B to A_*i*_
              Assign correct classification if contrast A_*i*_ most similar
              to equivalent contrast in B

The “most similar” is defined as the highest scoring map from the other group after scores are sorted by the absolute value in a descending fashion. By comparing the actual vs. the predicted label for each strategy for handling empty voxels, this evaluation can provide a straightforward assessment of the influence of empty voxels on image comparison Figure 1.

## 3. Results

### 3.1. Assessing the influence of thresholding

#### 3.1.1. Score distributions

Overall, both strategies to handle empty voxels (CCA and SVI) exhibited decreasing Pearson and Spearman similarity scores with increasing threshold, and this trend was prevalent whether the thresholding included both positive and negative values (Supplementary Video 1), or just positive values (Supplementary Video 2). For more highly correlated images, CCA seemed to inflate correlation scores. We observed that a group of more highly positive correlations present for CCA using positive and negative values is not present for CCA that includes only positive values. This suggests that using only positive values to calculate correlation serves to deflate scores (consistent with the fact that it is restricting the range of values), and using both positive and negative values inflates overall scores. It is not clear if this would be important for distinguishing contrasts of different types in the task of image comparison. It could be the case that “deactivations,” if they are non-task related will make two images more similar to one another, but in being consistent across tasks, will act as noise and decrease accuracy to distinguish different tasks and contrasts from one another. This finding has been suggested in recent work (see **Figure 4**) of Gorgolewski et al. (2015). When comparing CCA with SVI, the group of more highly positive values is relatively smaller, possibly due to the fact that the CCA reduces the size of the mask drastically, and the other strategies do not, for both positive and negative (Supplementary Image 1) and only positive values (Supplementary Image 2).

#### 3.1.2. Thresholding effects on classification accuracy

**Figure 2:**
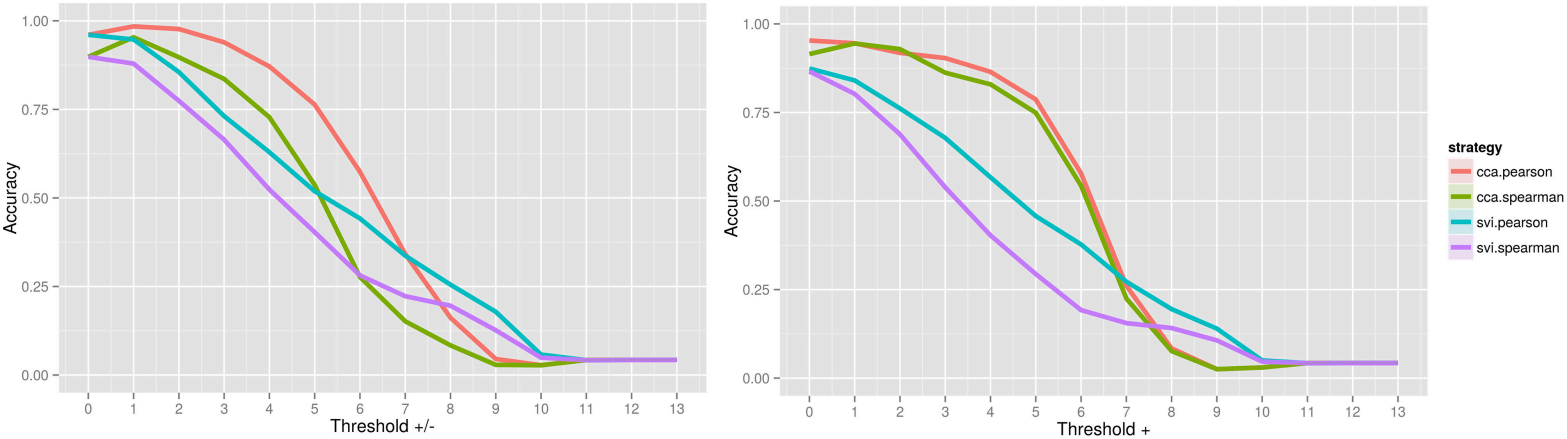
**Accuracy of image contrast classification at varying levels of image thresholding, for comparison of an unthresholded image against a set of images at each threshold, including positive and negative values (left) and positive values only (right)**. Complete case analysis (CCA) with a Pearson score had a maximum accuracy of 0.984 for a threshold of *Z* = ±1.0 (0.983, 0.985), outperforming single-value imputation (SVI).

**Figure 3:**
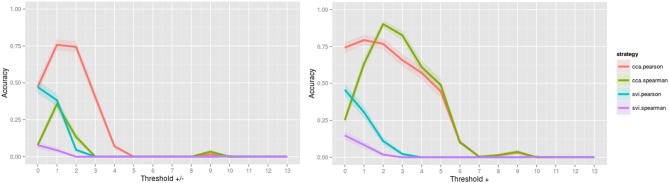
**Accuracy of image contrast classification at varying levels of thresholding, for the worst performing image, “0-back body,” from the working memory task**. Accuracy peaked at a threshold of *Z* = ±1.0 for complete case analysis with a Pearson score and at a threshold of *Z* = + 2.0 for complete case analysis with a Spearman score for each of positive and negative values (left) and positive values only (right).

When assessing the accuracy of image contrast classification at varying levels of image thresholding, CCA with Pearson has achieved the highest accuracy, followed by CCA with Spearman for both positive and negative values, and only positive values Figure 2. Accuracy peaked at 0.984 for a threshold of *Z* = ±1.0 (95% confidence interval, 0.983, 0.985) and at 0.953 for a threshold of *Z* = 0.0 (no threshold) (0.951, 0.954), a subtle indication that inclusion of positive and negative values improves accuracy of rankings globally. Interestingly, for image comparisons using positive and negative values, the maximum accuracy did not occur when comparing unthresholded to unthresholded images, suggesting that values close to 0 may serve as noise across all images and impede the classification task. When using a Pearson score for either directionality, a threshold value of *Z* = ±3.0 can be used to ensure minimally 0.90 accuracy in returning images of the same contrast, a threshold that corresponds with images having approximately only 25% of overlapping voxels within a standard brain mask (Supplementary Image 1). Investigation of the worst-performing contrast across folds (working memory task, contrast “0-back body,” accuracy = 0.758, standard deviation = 0.429) showed equivalent highest performance using CCA with a Pearson score at a threshold of *Z* = ±1.0 Figure 3, still much higher than chance (2%). Surprisingly, the global peak accuracy of 0.902 (95% confidence interval, 0.876, 0.928) occurred for a Spearman score with CCA using positive values only. Complete accuracy results for combined images across folds are included in Supplementary Data 2, and for the worst performing image in Supplementary Data 3.

#### 3.1.3. Image classification

**Figure 4:**
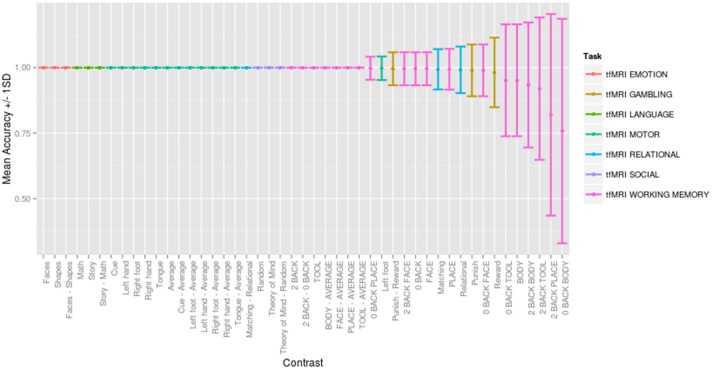
**Mean accuracy ±1.0 standard deviation for each contrast across 500 random folds for the optimally performing threshold (*Z* = ±1.0), direction (positive and negative), comparison strategy (complete case analysis) and similarity metric (Pearson score)**. Interactive confusion matrices for all thresholds, comparison strategies, and similarity metrics are available (http://vsoch.github.io/image-comparison-thresholding).

Across a range of thresholds, very high classification accuracy was achieved between contrasts, consistent with but substantially better than previous between-subject classification studies (e.g., Poldrack et al., 2009). Figure 4 presents the mean accuracy and standard deviation for each contrast across 500 random folds for the optimally performing threshold (*Z* = ±1.0), direction (positive and negative), comparison strategy (CCA) and similarity metric (Pearson score). Classification was consistently accurate to distinguish contrasts between tasks (with 30 contrasts being perfectly classified across all 500 folds), and classification errors were seen for similar contrasts within the same task (e.g., working memory contrasts “0-back body” vs. “body,” (overlapping conditions) and gambling task contrasts “punish,” vs. “reward.”) The only misclassification between tasks occurred for the gambling “punish - reward” contrast predicted to be the working memory “face - average” contrast. Interactive confusion matrices to explore the complete result are available (http://vsoch.github.io/image-comparison-thresholding).

## 4. Discussion

We have quantitatively assessed the impact of thresholding on performing pairwise image comparison with the two similarity metrics, Pearson and Spearman rank correlation coefficients. Our results suggest that a small amount of thresholding can improve image similarity computations. The Pearson metric using maps with both positive and negative values can be used to optimize classification of contrast maps, and including maps in the search set that have been thresholded up to *Z* = ±3.0 (corresponding to 25% of voxels non-empty within a standard brain mask overlapping between two images) ensures minimally 0.90 accuracy for retrieval of a map of the same contrast. Our results suggest that a minimum degree of thresholding (*Z* = ±1.0) can maximize accuracy of contrasts in a classification framework, and even moderate thresholding (*Z* = ±2.0) can increase accuracy as compared to comparison of unthresholded maps.

In assessing the distributions of Pearson and Spearman scores, we saw that including both positive and negative values inflated comparison scores for the higher correlations, however the overall distributions had generally deflated scores with increasing threshold. We suggest that this finding is a strength for the applied task of image comparison given the case that the highest subset of scores represent “truly similar” images. The image comparisons with lower correlations are likely driven by noise at small values, and so removing these values would deflate the overall score. However, studying these patterns in the distributions did not serve to answer the question of how classification accuracy is impacted by such thresholding, a question that was answered by our image classification task. In showing that inclusion of positive and negative values serves to improve accuracy of contrast classification, we suggest that negative and positive activations are both valuable sources of information, regardless of the subtle details about if scores are relatively inflated or deflated across our distributions. This improvement in accuracy could simply be due to the fact that a comparison is done with twice as many voxels, however this hypothesis does not hold true when comparing CCA to single value imputation. CCA, by way of being an intersection, included substantially fewer voxels than single value imputation, and was consistently more accurate Figure 2. Overall, our results support a decision to not arbitrarily exclude negative values when performing the task of image comparison. More work is needed to study the consistency, or variability, of these deactivations that have been sitting quietly in statistical brain maps before any consideration of eliminating them is to be done.

In a classification context, the scores themselves are almost irrelevant given that the images of the same contrast are returned, however this statement brings up a very basic question, “What is the quantitative language that we should use to compare two images?” We chose to define “similar” on a domain outside of the quantitative, namely, deriving maps from subjects performing the same behavioral tasks, solely because there is currently no answer to this question. Our analyses suggest that images thresholded up to *Z* = ±3.0 can be used to retrieve a corresponding contrast 9/10 times, and further, that images can be thresholded at *Z* = ±1.0 to maximize contrast classification performance.

Investigation of the worst performing contrast across folds revealed an interesting finding that using a Spearman score while including positive values only can increase classification accuracy by ~ 10% (for this single image). This particular image, “0-back body” from a working memory task, was most commonly misclassified as either “0-back” or “body” from the same task, an error that is likely attributable to the subtle differences between these contrasts. In retrospect, these contrasts are redundant. The contrast “0-back body” is a control condition for a working memory task that requires participants to respond if a body stimulus is presented (Owen et al., 2005), and so the contrast “0-back” is the generalization of this task over different stimulus types, and the contrast “body” combines all body conditions (“0-back” and “2-back”). Although these overlapping contrasts might have been eliminated from the classification task, they can be used as a case study for comparing two images with subtle differences. In this scenario, a strategy that would optimize classification of subtle differences might be used in combination with a strategy to optimize global accuracy (across tasks). Further, a finding like this questions the distinctness of these contrasts, and utility in deriving both. Finally, the inclusion of negative values hindering our ability to distinguish between these similar contrasts again questions the validity of these “deactivations” in the context of highly similar contrasts. An investigation of the value-added when including negative values for these highly similar contrasts is warranted.

Importantly, our results question two common opinions on thresholding in the neuroscience community. First, there is the idea that completely unthresholded maps are generally superior to thresholded images by way of providing more data. Our results suggest that voxels with very small values (for our dataset between *Z* = 0.0 and *Z* = ±1.0) primarily serve as noise, and analyses of unthresholded data may be negatively impacted by this noise.

Second, our results suggest that standard thresholding strategies, namely random field theory (RFT) thresholding, may not be optimal for subsequent image comparison because it eliminates subthreshold voxels with valuable signal. RFT requires a clusterforming threshold where only suprathreshold voxels are considered for further statistical analyses. For example, the popular neuroimaging software package FSL (Jenkinson et al., 2012) has a standard setting for a clusterforming threshold of *Z* = ±2.3 (*p* < 0.01), and the software SPM (Worsley, 2007) uses an even higher threshold of *Z* = ±3.1 (*p* < 0.001). To place this thresholding strategy in the context of our work, we generated thresholded maps using the FSL standard (*Z* = ±2.3) for a single subsample, including 47 contrasts for each of groups A and B, and compared the number of voxels within a standard brain mask for these maps compared to the optimal threshold for this data, *Z* = ±1.0 (Supplementary Data 4). We found that a threshold of *Z* = ±2.3 produces maps with on average 28.38% brain coverage (standard deviation = 16.1%), corresponding to an average decrease of 38.84% (standard deviation = 11.36%) in the number of brain masked voxels as compared to our maximum accuracy threshold of *Z* = ±1.0. This results in more sparse results, meaning producing maps with fewer voxels. Mapping this result into our accuracy space, a threshold of *Z* = ±2.3 corresponds with 96.56% classification accuracy, or a loss of ~1.86% accuracy for image classification as compared to our optimal. This percentage could be meaningful given a large database of images. A higher threshold (such as SPM's standard of *Z* = ±3.1) would result in a bigger loss of information and accuracy for image classification.

We have identified an optimal image comparison strategy in the context of the commonly practiced transformation of image thresholding. We did not test other transformations, and so we cannot confidently say that using this transformation of an image is the “best” strategy. While answering this question is outside of the scope of this paper, it is a question that is important to address if we aim to have consistent standards for reproducibility, and a common language for both humans and machines to understand image comparison. This strategy must be developed by first asking researchers what information is important for them to understand about the images (e.g., regional or spatial similarity, temporal similarity), what kind of noise is reasonable to expect given maps derived in subtly different ways, and then developing a set of standards to capture and balance those features. Finally, we are not claiming that there exists a global optimal threshold for such classification, but rather that researchers should take heed to identify optimal strategies for thresholding their datasets for use in a classification framework. The particular threshold values reported in this study likely depend on the quality of the data, the contrast to noise ratio, as well as the number of subjects, and thus are not directly applicable to new datasets.

### 4.1. Limitations

While our initial question was asking about filtering an image database based on thresholding (i.e., “What are the thresholds that can be included to ensure optimal classification of results?”), another interpretation of this analysis is about image transformations (i.e., “How far can we reduce an image and still get equivalent results?”). A limitation of this current work is that we did not test a more substantial set of image transformations. We also start with the basic assumption that, given that most images are unthresholded, and sharing of unthresholded images is the direction that the neuroimaging community is moving toward, a researcher would approach this task using an unthresholded map as a query image. Fair evaluation of classification accuracy to compare two thresholded maps would require a different approach that considers overlap between suprathreshold voxels in the case of small or non-existent overlap. Without carefully developed procedure to account for the sparse overlap, we would expect the classification accuracy to reduce dramatically. Due to this fact we recommend using fully unthresholded maps for sharing purposes (for example when uploading to repositories such as NeuroVault.org).

The image retrieval task using a statistical map constrained to an a-priori region of interest is a question not addressed by our work. The field of image based retrieval is fairly unexplored in context of statistical maps and some transformation of unthresholded maps might improve the classification performance. It could be that a transformation that weights voxels in an intelligent way, a binary metric, a region-based representation, or another dimensionality reduction algorithm would be optimal toward this goal. The use of an intersection mask between an unthresholded image A and thresholded image B also makes our metric asymmetric, and a symmetric metric to compare such maps might be desired. We are excited to investigate these ideas in future work.

The HCP data represents, to our knowledge, the largest publicly available dataset of single subject task data that allows for our analyses, and thus we are limited to making inferences based on this set of images. We recognize that these data are of higher quality than many other existing datasets, and it would be useful to compare the results to other datasets with many tasks across many subjects. These images shared acquisition parameters, voxel dimensions, and smoothing, and while it is relatively easy to transform images into a common space and size, we cannot predict deviances in our findings on different datasets. Finally, our analyses are focused on group statistical maps. Retrieval of contrast images for single-subject maps would be much more challenging due to the possibility of large inter-subject variability.

## 5. Conclusion

We have investigated the impact of thresholding on contrast classification, and suggested specific strategies to take when performing image comparison with unthresholded maps. This work is important, because image comparison with a result image as a query we believe will drive the next decade of meta-analysis. The applicability of our work is immediate as we have used our findings to drive the first version of our “image comparison” feature newly released in http://www.neurovault.org. While there are many questions to be investigated pertaining to the simple task of image comparison and the more complicated task of doing meta-analysis, this work is a first step toward deriving a holistic understanding of how to “best” compare the expanding landscape of publicly available statistical brain maps.

## Author contributions

The experiments were designed and interpreted by authors VS, KG, OK, JD, and RP. Analyses were carried about by author VS. Manuscript textual content was prepared by authors VS, OK, and RP, with substantial feedback from KG and JD. Figures, supplemental data, manuscript formatting, code, and web interfaces were produced by author VS. All authors approved of the final manuscript and are accountable for all aspects of the work.

## Funding

VS is supported by a William R. Hewlett Stanford Graduate Fellowship and a National Science Foundation Fellowship.

### Conflict of interest statement

The authors declare that the research was conducted in the absence of any commercial or financial relationships that could be construed as a potential conflict of interest.

## Supplementary material

Supplementary Video 1 **Similarity metric distributions**. Both strategies to handle empty voxels, including complete case analysis (CCA) and single value imputation (SVI), exhibited decreasing Pearson and Spearman scores as threshold increased when including both positive and negative values.

Supplementary Video 2 **Similarity metric distributions**. Both strategies to handle empty voxels, including complete case analysis (CCA) and single value imputation (SVI), exhibited decreasing Pearson and Spearman scores as threshold increased when including positive values only.

Supplementary Image 1 **Size of comparison set as a percentage of brain mask**. Complete case analysis (CCA) by way of including only voxels defined in both brain maps reduces the size of the mask more drastically than single value imputation (SVI) when including both positive and negative values.

Supplementary Image 2 **Size of comparison set as a percentage of brain mask**. Complete case analysis (CCA) by way of including only voxels defined in both brain maps reduces the size of the mask more drastically than single value imputation (SVI) when including positive values only.
