# Supplementary figures and images for: Effects of thresholding on correlation-based image similarity metrics
Source: Front Neurosci. 2015 Oct 29;9:418. doi: 10.3389/fnins.2015.00418 (PMC4625081; doi:10.3389/fnins.2015.00418)

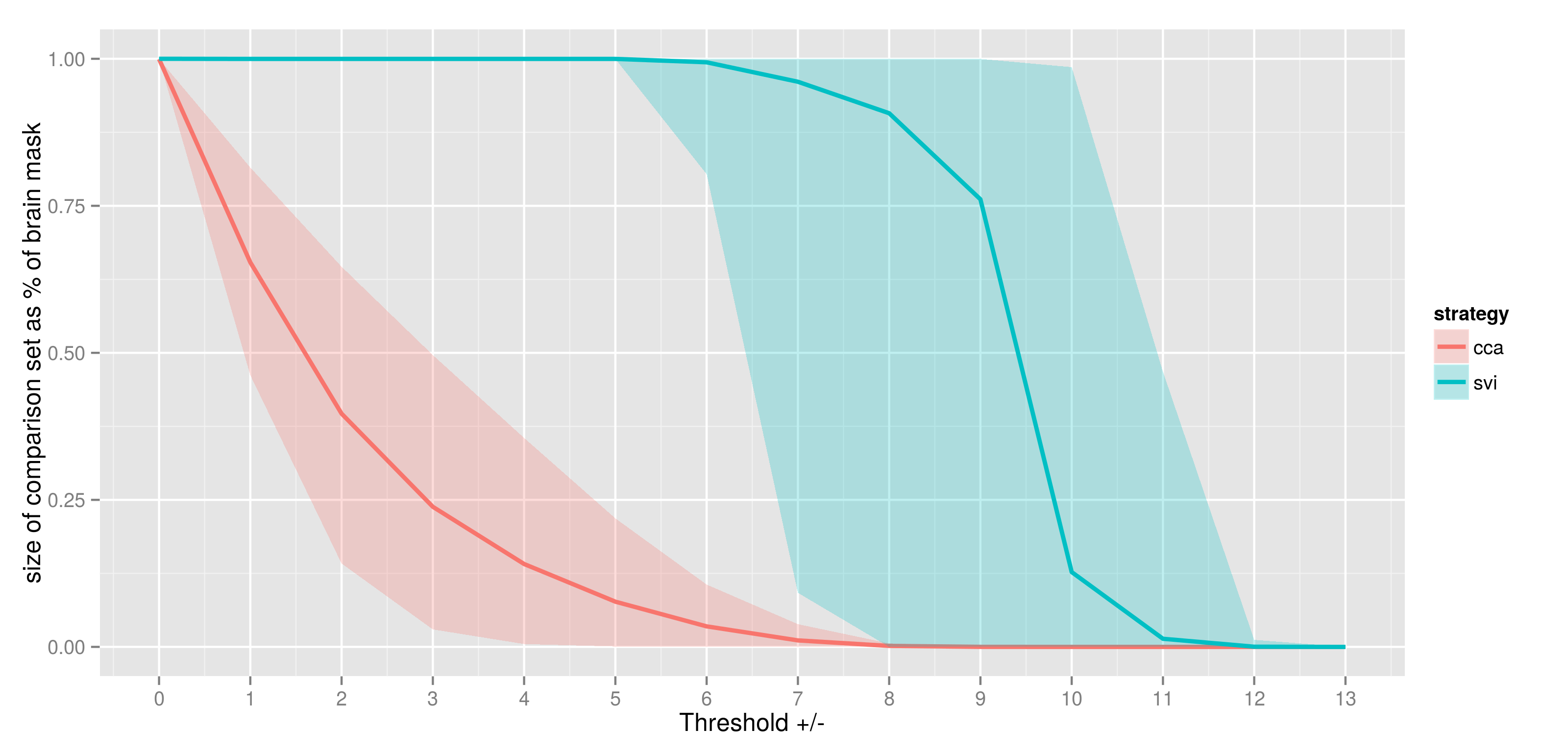

Supplement: Supplementary Image 1 — Size of comparison set as a percentage of brain mask. Complete case analysis (CCA) by way of including only voxels defined in both brain maps reduces the size of the mask more drastically than single value imputation (SVI) when including both positive and negative values. [file Image1.PNG]

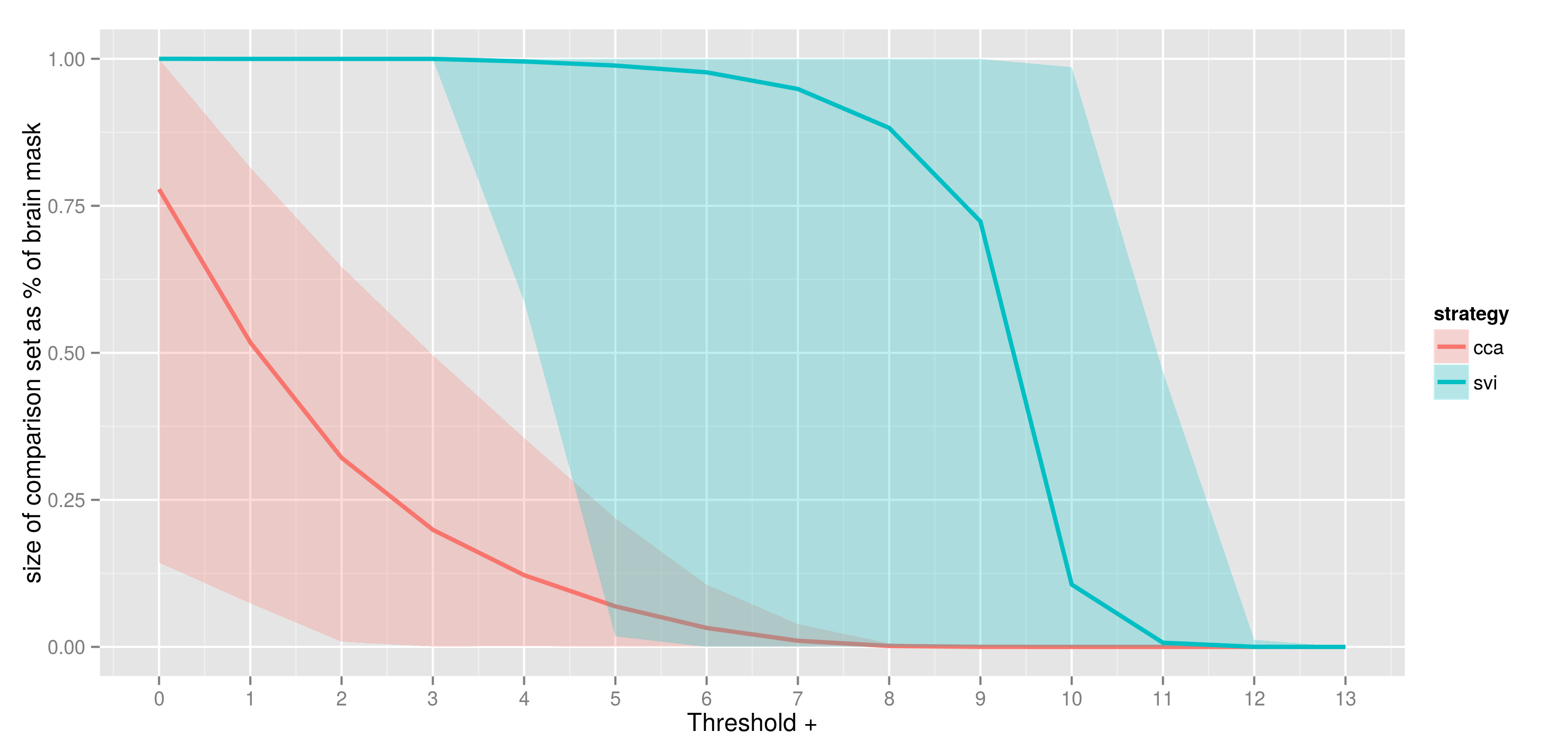

Supplement: Supplementary Image 2 — Size of comparison set as a percentage of brain mask. Complete case analysis (CCA) by way of including only voxels defined in both brain maps reduces the size of the mask more drastically than single value imputation (SVI) when including positive values only. [file Image2.PNG]
